# Assessment of Medication Use among University Students in Ethiopia

**DOI:** 10.1155/2017/4530183

**Published:** 2017-03-14

**Authors:** Dessalegn Asmelashe Gelayee, Gashaw Binega

**Affiliations:** ^1^Department of Pharmacology, College of Medicine and Health Sciences, University of Gondar, Gondar, Ethiopia; ^2^Department of Clinical Pharmacy, College of Medicine and Health Sciences, University of Gondar, Gondar, Ethiopia

## Abstract

*Background.* The extent, nature, and determinants of medication use of individuals can be known from drug utilization studies.* Objectives.* This study intended to determine medication consumption, sharing, storage, and disposal practices of university students in Northwest Ethiopia.* Methods.* A descriptive cross-sectional study was conducted on 404 university students selected through stratified random sampling technique. Data were collected using self-administered questionnaire and analyzed with SPSS version 20 statistical software. Pearson's Chi-square test of independence was conducted with *P* < 0.05 taken as statistically significant.* Results.* At 95.3% response rate, the prevalences of medication consumption and sharing were 35.3% (*N* = 136) and 38.2% (*N* = 147), respectively. One hundred (26%) respondents admitted that they often keep leftover medications for future use while the rest (*N* = 285, 74%) discard them primarily into toilets (*N* = 126, 44.2%). Evidence of association existed between medication taking and year of study (*P* = 0.048), medication sharing and sex (*P* = 0.003), and medication sharing and year of study (*P* = 0.015).* Conclusion.* There is a high prevalence of medication consumption, medication sharing, and inappropriate disposal practices which are influenced by sex and educational status of the university students. Thus medication use related educational interventions need to be given to students in general.

## 1. Introduction

Irrational use of medicines is a global problem and there are many ways leading to this such as patient usage of too many medicines, inadequate dosage of antibiotics, use of antibiotics for nonbacterial infections, overuse of injections when oral medication can be more appropriate, prescribing inappropriate medicines to clinical guidelines, and self-medication [1]. Thus, the World Health Organization (WHO) reported that 50% of all medicines are prescribed, dispensed, or sold incorrectly and 50% of patients do not take their medicines satisfactorily [2]. Currently, there is a global increase in the consumption and spending of drugs and by the year 2016 annual global spending on medicines was expected to reach nearly $1.2 trillion [3]. In Ethiopia, the annual pharmaceutical market is estimated to be worth US$ 400 to 500 million and is growing at a rate of 25% per annum. It is believed to reach an approximate value of just under US$ 1 billion by 2018 [4]. In many developing countries including Ethiopia, drugs constitute 20–40% of healthcare budgets [5]. The nation's healthcare system still suffers from limited availability of health resources, overreliance on out-of-pocket payments, and inefficient and inequitable use of resources. Households' out-of-pocket expenditure accounts for 47% of the total drug expenditure [6]. Therefore, very significant savings can be made by promoting appropriate drug use. To make this happen, drug utilization studies are very essential as they can describe the extent, nature, and determinants of drug use by individuals [7]. Medication use is undoubtedly a crucial issue in colleges and among young adults, including medical or nonmedical use of prescription and nonprescription drugs [8]. In addition, medications have been detected throughout the environment, many resulting from improper patient disposal of unused pharmaceuticals via environmentally unfriendly routes, such as the sink, toilet, or rubbish bin. In some cases, they have been shown to have a detrimental effect [9]. It is worth noting that some medications can cast effects on the environment (such as on bacteria and animals) well below the concentrations that are usually used in safety and efficacy studies [10]. The first step to correct irrational use of medicines and/or to evaluate previous interventions is to measure and characterize it. Therefore, this study was conducted to assess drug consumption and use behavior of college students so that the finding can be used to design further studies and interventions.

## 2. Methods

This cross-sectional study was done from February to June 2014 to assess medication use practice of the social science students of University of Gondar, Northwest Ethiopia. The sample size was determined using a formula of *n* = *z*^2^*p*(1 − *p*)/*w*^2^ and 5% contingency with the following assumption: a *P* value of 0.05, *z* = 1.96 and CI = 95%, *w* = 0.05. Thus, 404 students were obtained using a stratified random sampling technique based on sex and educational status. A self-administered questionnaire was developed based on previous studies, pretested, and then used to collect data. The survey instrument consisted of questions regarding sociodemography, medication consumption during the study time, medication sharing practice, drug storage, and disposal practice as well as sources of medicines and medicine related information. Data were analyzed using SPSS version 20 statistical software and Pearson's Chi-square test of independence was conducted at *P* ≤ 0.05 taken as statistically significant. Ethical clearance was issued from the Department of Pharmacology, College of Medicine and Health Science, University of Gondar, and informed consent was obtained from the participants.

## 3. Results

**Figure 1:**
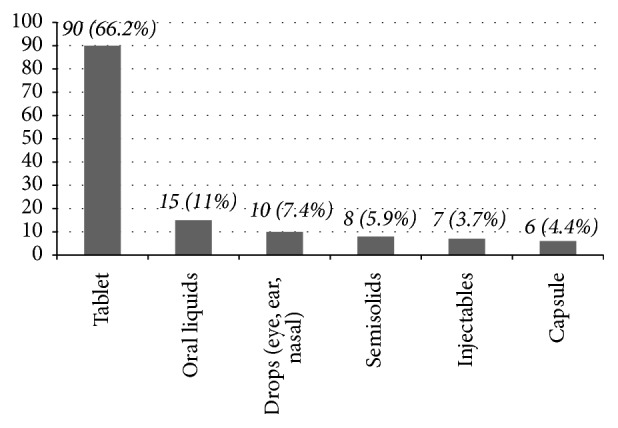
Dosage forms taken by the respondents (*N* = 136). About 136 respondents were taking medications at the study time in the form of tablets (*N* = 90), oral liquid (*N* = 15), eye-ear-nasal drop (*N* = 10), and semisolid (*N* = 7) and injectable (*N* = 6) medicines. Multiple response variable.

**Figure 2:**
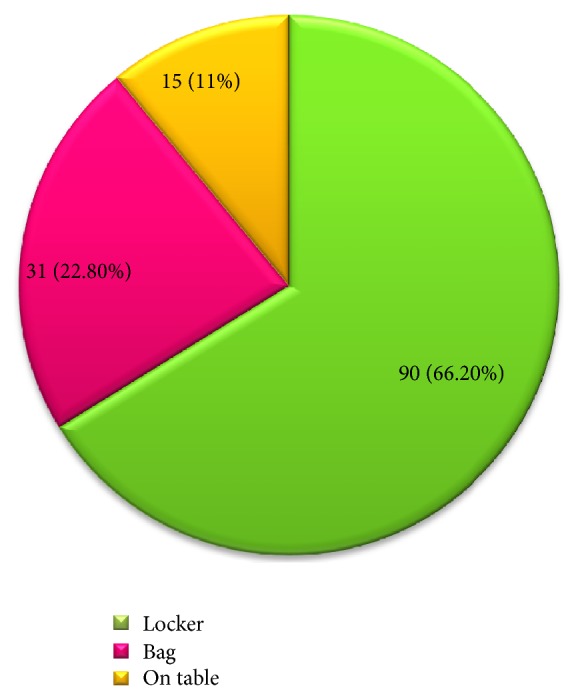
Medication storage practice of respondents (*N* = 136). From 136 respondents who were taking medications, 66.2%, 22.8%, and 11% respondents, respectively, stored their medication in lockers, bags, and on open tables.

**Figure 3:**
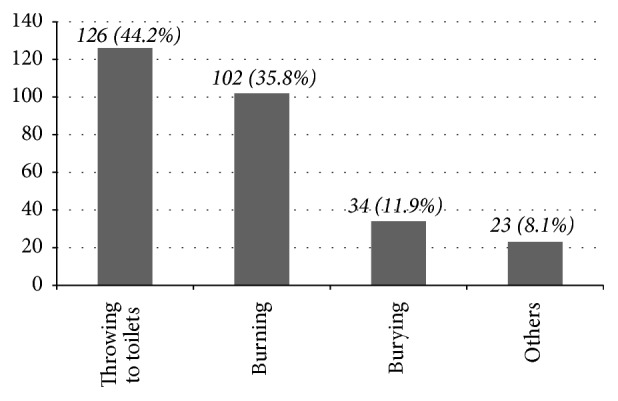
Respondents' disposal systems of leftover medicines (*N* = 285). From 285 respondents who used to discard leftover medications, 126, 102, and 34 students, respectively, discard them into toilets and through burning and burying. About 23 students throw them to open fields or dust bins. Others include throwing to open field and putting in dust bins.

Three hundred and eighty-five non-healthcare college students completed questionnaires in this study and the majority were male (55.8%), below third year (59.5%), and with monthly average income of 200–500 Eth birr (64.7%) (Table 1). The prevalence of medication consumption at the study time was 35.3% (*N* = 136). As shown in Figure 1, most (*N* = 90, 66.2%) of the respondents were taking tablets and oral liquid formulations (*N* = 15, 11%). Medication storage practices of 136 respondents who were taking medications at the study time were assessed. As shown in Figure 2, medications were kept in a secured place mainly inside lockers. When asked about the fate of leftover medicines, 100 (26%) of the 385 respondents often keep them for future use while the majority (*N* = 285, 74%) discard them. The main disposal approach for leftover medicines were throwing into toilets (*N* = 126, 44.21%) and burning (*N* = 102, 35.79%) (Figure 3).

One hundred and forty-seven (38.2%) of the respondents shared medicines with other people in the past 6 months. Type of medications shared were prescribed (*N* = 62, 42.2%), nonprescribed (*N* = 39, 26.5%), and both types of medications (*N* = 46, 31.3%). As shown in Table 2, 203 (52.7%) and 93 (24.2%) respondents, respectively, identified public pharmacies and leftover medicines as their primary sources of medicines. Medicine related information was received from relatives and friends as well as health professionals. The sex of the respondent has influence on medication sharing practices [male = 68 (31.6%), female = 79 (46.5%); *P* = 0.003] while students' educational status influences both medication consumption [≤year 2 = 90 (39.3%), ≥year 3 = 46 (29.5%); *P* = 0.048] and sharing behavior [≤year 2 = 76 (33.2%), ≥year 3 = 71 (45.5%); *P* = 0.015] (Table 3).

**Figure 4:**
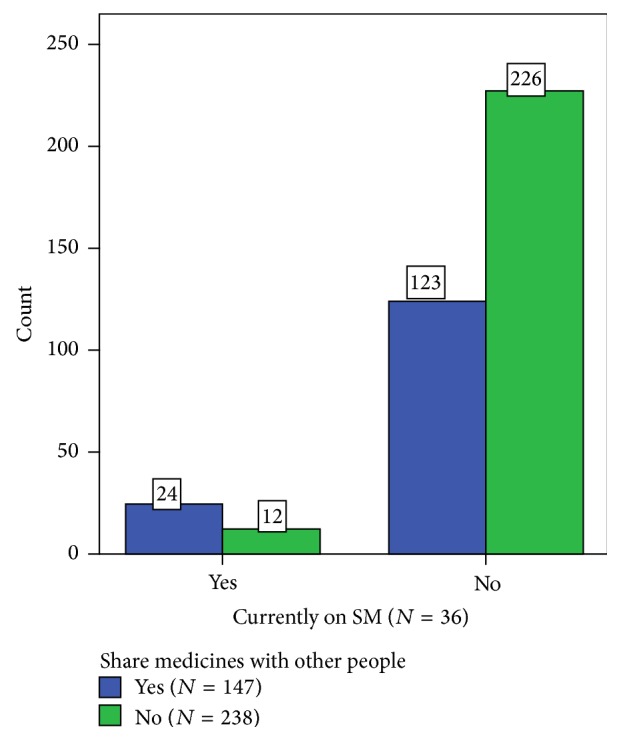
Association between respondents' practice of self-medication and sharing medications with other people. About 66.7% of self-medication practicing respondents share medications compared to 35.2% of respondents not practicing self-medication at the study time (*X*^2^ = 13.652, *P* < 0.001).

**Figure 5:**
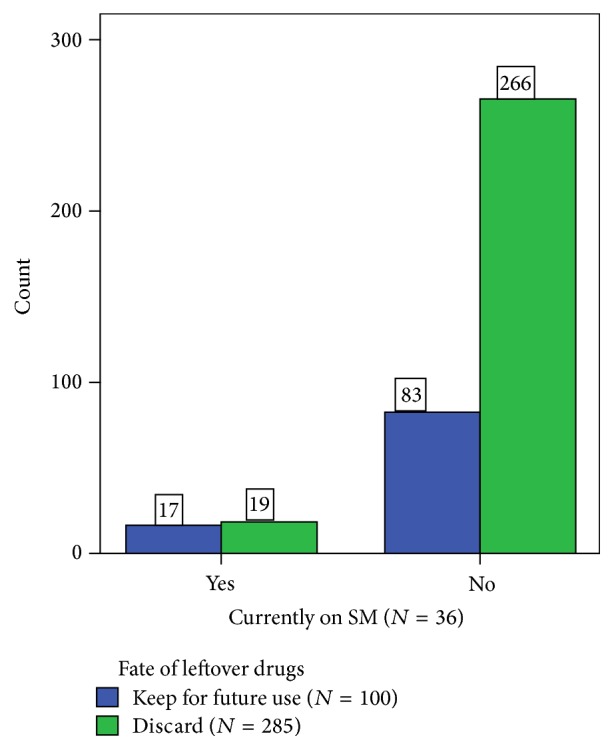
Association between respondents' practice of self-medication and keeping leftover medications for future use. About 47.2% of self-medication practicing respondents kept leftover medications for future use compared to 33.3% of respondents not practicing self-medication at the study time (*X*^2^ = 9.325, *P* = 0.002).

As can be seen in Figures 4 and 5, the presence of self-medication practice is associated with sharing medications with other people (*X*^2^ = 13.652, *P* < 0.001) and not discarding leftover medications (*X*^2^ = 9.325, *P* = 0.002).

## 4. Discussion

There was a high prevalence (*N* = 129, 33.5%) of medication consumption at the study time and in this context it is crucial to ascertain appropriate drug use in this population. Such high use of medications at a given time has alarming health and economic implications. Therefore, further studies are important to address determinants of health and patterns of drug use among college students. In the present study, medication intake was associated with students' educational status (*P* = 0.048). It may be assumed that more junior students may suffer from illnesses more than their seniors because of less adaptation to the environment and more stress in their study. Worldwide, tablets are the most widely used dosage forms because of self-administration and ease in manufacturing [11]. Thus, majority (*N* = 90, 66.2%) of medication consumers in the present study were taking tablets. It is encouraging that most (*N* = 90, 66.2%) students who were taking medications used locked cupboards to store medications. This finding is far better than that reported by Matson et al. where 77% college students in South Africa never store medications in a locked place [12].

More than a quarter of respondents often keep leftover medicines for future use and this is unacceptable practice since reuse of some prescribed medications may not guarantee medication safety. Previous studies in Ethiopia and abroad reported that leftover medications may be used for self-medication practice [12, 13]. The unused medications may also be a threat for suicidal attempts given that college students may experience day-to-day difficulties as a result of living away from home and study pressure. Throwing leftover medicines into toilets and burning were identified as major disposal approaches. This strengthens a report by Azad et al. that college students disposed of unwanted medications into toilet and landfills [14]. Both methods of drug disposal (flushing into toilets and burning) are not the methods of choice as it is possible for both to introduce pharmaceutical residues into the natural environment and pose a significant environmental risk [15]. As burning medications potentially decomposes pharmaceuticals to inactive chemical residues, further studies should be carried out to determine whether the method of burning reported by participants is effective and whether these processes pose less environmental risk than flushing medicines into the toilet. However, returning unused medications into nearby public pharmacies may be a better way of disposing them. Such practices are more commonly used worldwide [16] and establishing such systems needs to be given attention in Ethiopia as well.

There is a high prevalence (*N* = 147, 38.2%) of medication sharing practice by the respondents within the previous 6-month recall period which is associated with sex (*P* = 0.003) and educational level of students (*P* = 0.015). Medication sharing is not without risks and it may result in delays in seeking professional help, misdiagnosis, development of antibiotic resistance, possible complications from drug interactions, and increased risk of side effects [17]. Goulding et al. reported a 32% lifetime prevalence of sharing prescription medication among Irish college students [18]. As such, the prevalence of medication sharing in the present study is very high as it is based on a 6-month recall period. A better public health education emphasizing the risks associated with sharing medications, particularly within the Ethiopian College system, is needed.

Leftover medicines were identified as the usual source of medicine for a quarter of the respondents in the present study. However, using them for new illnesses has a potential to do harm as there may be wrong diagnosis illnesses by the students or medications may be contraindications. Thus, precaution is needed. Most respondents (*N* = 138, 35.84%) relied on relatives and friends for medicine related information probably because of ease of access to them. Only 125 (32.47%) of the total respondents communicate with health professionals. Thus, further studies are needed to identify and overcome the communication barriers.

## 5. Conclusion

The findings from the present study revealed a high prevalence of medication consumption, medication sharing practices, and inappropriate medication disposal practice among university students. These outcomes were influenced by sex and educational status as well as self-medication practice by the students. Thus, public health education should be given to students in general regarding appropriate drug use and drug takeback systems shall be introduced.

## Figures and Tables

**Table 1:** Sociodemography of respondents.

Variable	*N* (%)
Sex	
Male	215 (55.8)
Female	170 (44.2)
Year of study	
First year	116 (30.1)
Second year	113 (29.4)
Third year	130 (33.8)
Fourth year	12 (3.1)
Fifth year	14 (3.6)
Monthly income	
<200 Eth birr	80 (20.8)
200–500 Eth birr	249 (64.7)
>500 Eth birr	56 (14.5)

1 USD = 19.95 Eth birr.

**Table 2:** Sources of medicine and medicine related information (*N* = 385).

Variable	Responses	
	*N*	%
Usual source of medicines		
Government pharmacy	203	52.7
Private pharmacy	142	36.9
From previous leftover drugs	93	24.2
Relatives and friends	26	6.8
Supermarket	9	2.3
Sources of information on medicines		
Relatives and friends	138	35.8
Health professionals	125	32.5
Media advertisement	104	27
Internet	74	19.2
Textbooks	53	13.8
Drug leaflet	41	10.7

NB: multiple answers for both variables.

**Table 3:** Association between independent and dependent variables (*N* = 385).

Dependent variable	Independent variable		
	Sex	Study year	Monthly income
Taking medications at the study time (136)	Male (*N* = 70, 32.6%)	≤year 2 (*N* = 90, 39.3%)	<200 birr (*N* = 28, 35%)
	Female (*N* = 66, 38.8%)	≥year 3 (*N* = 46, 29.5%)	200–500 birr (*N* = 82, 32.9%) >500 birr (*N* = 26, 46.4%)
	*X* ^2^ = 1.631, *P* = 0.202	*X* ^2^ = 3.912, *P* = 0.048^*∗*^	*X* ^2^ = 3.650, *P* = 0.161

Fate of leftover drug is discard (285)	Male (discard =162, 75.3%)	≤year 2 (*N* = 168, 73.4%)	<200 birr (*N* = 64, 80%)
	Female (discard = 123, 72.3%)	≥year 3 (*N* = 117, 75%)	200–500 birr (*N* = 182, 73.1%) >500 birr (*N* = 30, 53.5%)
	*X* ^2^ = 0.443, *P* = 0.506	*X* ^2^ = 0.129, *P* = 0.719	*X* ^2^ = 2.157, *P* = 0.340

Share medications with others (147)	Male (*N* = 68, 31.6%)	≤year 2 (*N* = 76, 33.2%)	<200 birr (*N* = 26, 32.5%)
	Female (*N* = 79, 46.5%)	≥year 3 (*N* = 71, 45.5%)	200–500 birr (*N* = 93, 37.3%) >500 birr (*N* = 28, 50%)
	*X* ^2^ = 8.861, *P* = 0.003^*∗*^	*X* ^2^ = 5.972, *P* = 0.015^*∗*^	*X* ^2^ = 4.481, *P* = 0.106

1 USD = 19.95 Eth birr. *∗* refers to statistical significance at *P* < 0.05.
